# Quantifying myosin light chain phosphorylation in single adherent cells with automated fluorescence microscopy

**DOI:** 10.1186/1471-2121-8-43

**Published:** 2007-10-17

**Authors:** Kiran Bhadriraju, John T Elliott, My Nguyen, Anne L Plant

**Affiliations:** 1 SAIC, Arlington, VA, USA; 2 Cell and Tissue Measurements Group, Biochemical Sciences Division, National Institute of Standards and Technology, Gaithersburg, MD, USA; 3 American University, Washington DC, USA

## Abstract

**Background:**

In anchorage dependent cells, myosin generated contractile forces affect events closely associated with adhesion such as the formation of stress fibers and focal adhesions, and temporally distal events such as entry of the cell into S-phase. As occurs in many signaling pathways, a phosphorylation reaction (in this case, phosphorylation of myosin light chain) is directly responsible for cell response. Western blotting has been useful in measuring intracellular phosphorylation events, but cells are lysed in the process of sample preparation for western blotting, and spatial information such as morphology, localization of the phosphorylated species, and the distribution of individual cell responses across the population is lost. We report here a reliable automated microscopy method for quantitative measurement of myosin light chain phosphorylation in adherent cells. This method allows us to concurrently examine cell morphology, cell-cell contact, and myosin light chain diphosphorylation in vascular smooth muscle cells.

**Results:**

Paraformaldehyde fixation and Triton X-100 permeabilization preserved cell morphology and myosin light chain phosphorylation better than the alternative fixation/permeabilization methods tested. We utilized automated microscopy methods to acquire three color images, determine cell spread area, and quantify the intensity of staining within each cell with anti-phospho-MLC antibody. Our results indicate that A10 rat aortic smooth muscle cells exhibit a re producible non-Gaussian distribution of MLC phosphorylation across a population of unsynchronized genetically identical cells. Adding an inhibitor of Rho kinase, Y27632, or plating cells on a low density of fibronectin, reduced phospho-myosin light chain signal as expected. On the other hand, adding calyculin A, an activator of contractility, increased myosin light chain phosphorylation. The IC_50 _for myosin light chain phosphorylation using Y27632 was determined to be 2.1 ± 0.6 micrometers. We observed a positive linear relationship between cell area and myosin light chain diphosphorylation, which is consistent with what has been reported in the literature using other methods.

**Conclusion:**

Our results show that using proper specimen fixation techniques and background subtraction methods, imaging cytometry can be used to reliably measure relative myosin light chain phosphorylation in individual adherent cells. Importantly, the ability to make this measurement in adherent cells allows for simultaneous measurement of and correlation with other parameters of cellular topography such as morphology and cell-cell proximity. This assay has potential application in screening for drug development.

## Background

Analysis of signaling events within individual cells with microscopy imaging has several advantages over non-imaging techniques. The principal advantage of imaging cytometry over plate reader assays and even other single cell techniques such as flow cytometry is the potential for collection of morphological and spatial information over subcellular and supracellular length scales [1]. This can be an advantage for cell based assays for drug cytotoxicity screening where even relatively simple morphological measurements, such the area of the nucleus, have been shown to be predictive of clinical outcome [2]. Imaging cytometry can collect spatial information on several hundreds of adherent cells, as well as provide signal quantification in single adherent cells and quantification of differences between cells in the population. Image cytometry has been used to study activation of signaling proteins such as p38 MAP kinase where change in localization of the protein from the cytoplasm to the nucleus has been used to score activation [3].

Artifacts introduced during sample fixation and immunostaining can compromise the cellular location and amount of proteins of interest. Samples for end-point imaging cytometry are typically fixed using a crosslinking or denaturing fixative such as paraformaldehyde, or various solvents such as methanol and ethanol [4]. Fixation methods for quantitative immunofluorescence labeling may need to be tailored for different targeted proteins [5] in order to stabilize relevant cell structures, to enable epitope exposure and to preserve signal intensity. The measurement of phosphorylated proteins in cells by microscopy presents particular challenges because the phosphorylation states are dynamic and sensitive to cellular phosphatases [5]. Establishing and validating the appropriate fixation and staining procedures for maintaining amount and location of antigen target is critical for quantitative microscopy applications.

In anchorage dependent cells, myosin-generated contractile forces on the extracellular matrix (ECM) underpin several vital cellular processes related to cell adhesion. These include rapid events such as the formation of stress fibers and focal adhesions [6], and temporally distal events such as those regulating entry in to S-phase [7,8]. Hence, tools to measure myosin activity are of particular interest in cell adhesion and cytoskeleton research. The phosphorylation of myosin on the 20 kD regulatory light chain (MLC) is mainly responsible for changes in its activity. MLC can be monophosphorylated on Ser19, or diphosphorylated on Ser19/Thr18. There are multiple pathways for the activation of myosin light chain phosphorylation including p21 activated kinase (PAK, [9], myosin light chain kinase (MLCK, [10]), and Rho-dependent kinase (ROCK) [11] and drugs that inhibit disregulated contractility are being investigated as potential therapeutics for pulmonary hypertension [12]. Since the activation of myosin is purported to be critical to a number of important downstream processes including cell migration, proliferation and transformation [13,14], a reliable microscopy based MLC phosphorylation assay could provide valuable insight into these processes. In this study, we developed and optimized a protocol for using automated fluorescence microscopy to quantify the level of MLC diphosphorylation in adherent cells.

## Results

To take advantage of the benefits of microscopy, it was important to preserve the overall morphology of the specimen while also obtaining an optimal signal for myosin light chain phosphorylation. We first used overall cell morphology as the qualitative criteria to screened several fixation techniques. We tested four different fixation reagents: ethanol (EtOH), methanol (MeOH), acetone (Ac), and paraformaldehyde (PFA). PFA best preserved cell morphology while the organic solvents ethanol, methanol, and acetone, gave variable results as fixatives and in some cases caused cells to round up upon addition of any one of the three solvents (data not presented). PFA was selected as the fixation reagent, and four different permeabilization methods were screened to select the optimal permeabilization strategy. After fixation by PFA at room temperature, cells were permeabilized with methanol (PFA+MeOH, 4°C), ethanol (PFA+EtOH, 4°C), acetone (PFA+Ac, 4°C) or triton X-100 in PBS (PFA+TX100, room temperature). We also tested a simultaneous fixation and permeabilization procedure by including 0.5% triton X-100 in the PFA solution (PFATX, room temperature). The presence of phospho-MLC antibody staining along well-defined f-actin stress fibers was used as the criterion for evaluating the permeabilization method.

**Figure 1:**
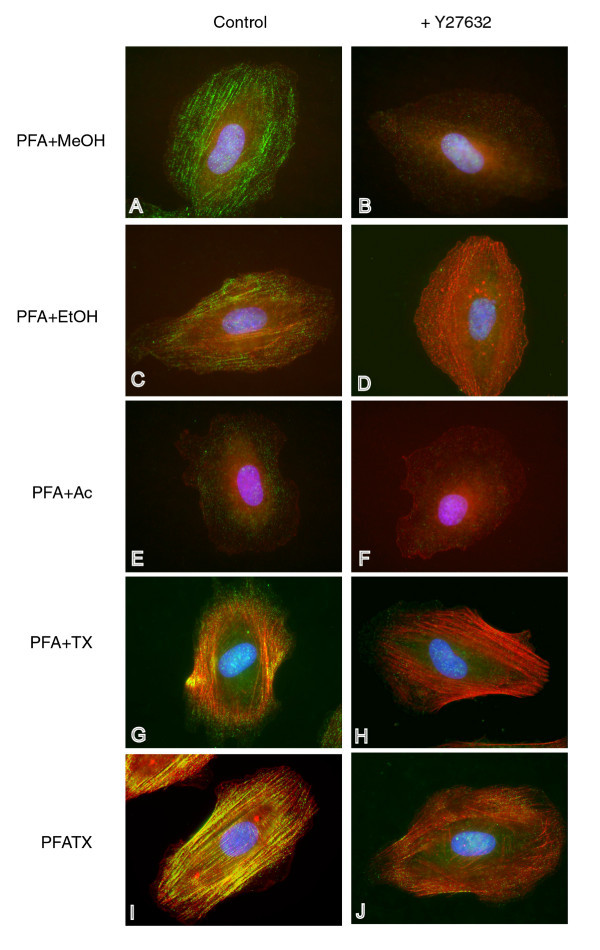
**Results of fixing and permeabilizing conditions on phospho-MLC and actin filament staining**. Images in the left column show cells that were fixed in the absence of any drugs to inhibit MLC phosphorylation. Images on the right depict cells fixed after treatment with 10 μM Y27632 to inhibit MLC phosphorylation. Fluorescence from phalloidin (which stains actin filaments) is indicated by red, anti-diphospho-MLC fluorescence by green, DAPI staining of nuclear DNA by blue, yellow indicates co-localization of red and green signal, pink indicates co-localization of red and blue signal.

Each of the different permeabilization techniques resulted in distinct differences in the staining patterns. MeOH permeabilized cells showed long filaments of phospho-MLC staining organized in a manner reminiscent of f-actin stress fibers, but very low intensity f-actin stress fiber staining by Alexa 594-phalloidin (Fig. 1A,B). EtOH permeabilized cells showed an expected pattern of staining typical of both phospho-myosin filaments and f-actin stress fibers but did not produce the same intensity of staining as the PFATX method (Fig. 1C,D), while acetone permeabilized cells did not show staining for either phospho-MLC or stress fibers (Fig. 1E,F). Longer exposure times were required for the Alexa 594-phalloidin images for cells permeabilized with MeOH (10 seconds), EtOH (10 seconds), and Ac (5 seconds) than for cells permeabilized with PFA+TX or PFATX (1 second). The two TX-100 permeabilization methods yielded comparable stress fiber morphology (Fig. 1G,I), but PFATX resulted in the best overall staining of stress fibers and phosphorylated MLC (Fig. 1I), and the greatest fold change in MLC-phosphorylation signal upon inhibition by Y27632 (data not presented). In each of these fixation conditions where phospho-MLC signal could be observed, treatment of the cells with 10 μM Y27632 for 1 h before fixation dramatically reduced the signal from phospho-MLC (Fig. 1B,D,H,J). Upon Y27632 treatment, even when most of the MLC phosphorylation signal was inhibited, there still remained well organized parallel strands of f-actin (compare Fig. 1I to 1J). Based on these results, PFATX was found to be optimal among the methods tested to preserve myosin light chain phosphorylation and actin stress fibers.

We quantified myosin light chain phosphorylation intensity in individual cells by employing Texas-Red maleimide (Tx-Red) labeling of each cell as a mask for the entire cell area [15], and applying that mask to the same field examined with appropriate filters for Alexa 488 immunofluorescence. We found no systematic contribution to the phospho-MLC signal from the Tx-Red cell mask as discussed in Methods. We examined the contribution of non-specific binding of secondary antibody to the measured phospho-MLC signal and found a small constant contribution to the signal in secondary antibody only controls. This background signal was corrected for as described in Methods.

**Figure 2:**
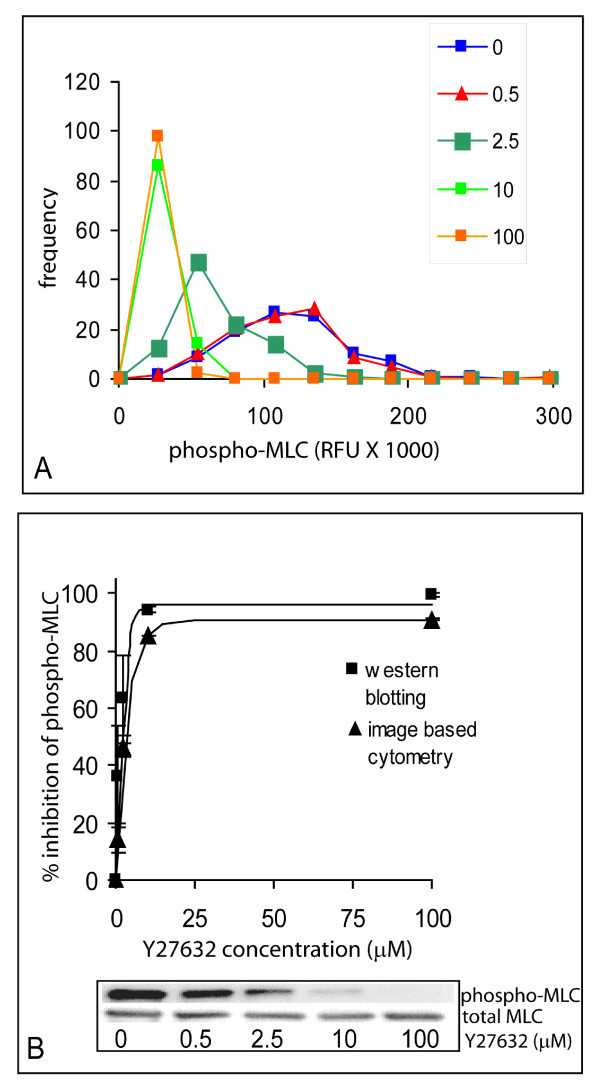
**Dose response of phospho-MLC to Y27632 concentration**. A) MLC diphosphorylation decreased with increasing concentration of Y27632 as shown by representative histograms of the range of  phospho-MLC fluorescence in 150 cells measured. Histograms labels refer to concentration (μM) of Y27632 added. B) Percentage inhibition of MLC-phosphorylation obtained by quantitative light microscopy and by Western blotting, plotted as a function of Y27632 concentration. The curves were fit to an exponential function as described in Materials and Methods to obtain an IC_50 _of 2.1 ± 0.6 μM by quantitative imaging and 1.3 ± 1.2 μM by Western blotting. Error bars indicate standard deviations of 3 replicate experiments. Representative images of the western blots used to quantify phospho-MLC, total MLC, and the concentrations of Y27632 used are shown below the graph.

Figure 2A shows the effect of increasing concentrations of the ROCK inhibitor on the intensity of phospho-MLC staining. The amount of diphospho-MLC in cells decreased with increasing concentration of Y27632. By examining individual cells, it is clear that MLC diphosphorylation varies between individual cells in the population. At 10 μM of Y27632, a concentration which is commonly reported in literature for use with cells, about 85% of the cellular diphospho-MLC was inhibited (Fig. 2B). Using the means of the distributions, the Y27632 concentration for half maximal inhibition of phospho-MLC, or IC_50 _value, was estimated from the inhibition curve to be 2.1 ± 0.6 μM (n = 3, Fig. 2B). This result was not significantly different from that obtained by Western blotting, 1.3 ± 1.2 μM (n = 3, Fig. 2B).

**Figure 3:**
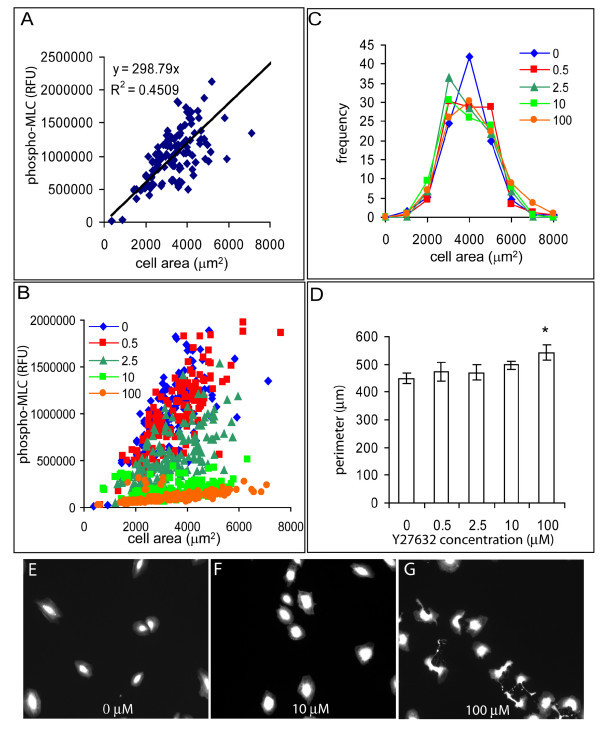
**The relationship between cell shape and phospho-MLC in individual cells**. A) In the absence of any drug treatment, the area of individual cells was linearly correlated with total diphospho-MLC content per cell. The data shown are from a representative experiment indicating measurements on about 150 cells; RFU (relative fluorescent units) B) Inhibiting ROCK by the addition of the indicated concentrations of Y27632 inhibited phospho-MLC in individual cells while preserving the linear relationship between cell spreading and MLC diphosphorylation. Representative data are shown from experiments involving about 150 cells. C) Treatment with Y27632 did not change the distribution of cell areas within the population as indicated by the similarity of the histograms. D) Inhibiting ROCK increased the perimeter of the cells compared to no drug treatment. These data are average values of cellular perimeters determined from three replicate experiments. Statistically significant difference compared to the perimeter of single cells in the absence of inhibitor (p < 0.05) is indicated by '*'. E) Representative cells in the absence of Y27632, F) in the presence of 10 μM Y27632 and G) 100 μM Y27632. Cells were stained with Tx-Red maleimide as described in Methods.

Using Tx-Red staining of cells to determine the spread area of individual cells, we examined the relationship between phospho-MLC intensity and cell morphology. We found a correlation between cell area and myosin light chain diphosphorylation (Fig. 3A) indicating that larger cells tended to contain more activated MLC. When cells were treated with Y27632, the diphospho-MLC signal was decreased, but the relation between MLC phosphorylation and cell area remained linear (Fig 3B) over the full range of cell areas. We also observed no significant change in the mean area or the distribution of cell areas measured across the population in absence or presence of Y27632 (Fig 3C). The mean cell areas and their standard deviations from three replicate measurements were 3465.4 ± 15.7 μm^2 ^in the absence of Y27632 and 3523.6 ± 90.6 μm^2^, 3877.4 ± 55.04 μm^2^, 3673.1 ± 193.1 μm^2^, and 3505.8 ± 63.3 μm^2 ^in the presence of 0.5, 2.5, 10 and 100 μM Y27632 respectively. The coefficient of variation (CV) of cell sizes within the population was also measured for each treatment, in order to assess the range of cell sizes present. The CVs were calculated from the standard deviation divided by the mean cell area within the population. Standard deviations between CVs determined from three replicate experiments were also calculated. The CV in cell area in the absence of Y27632 was 50.4 ± 11.2 and was 49.2 ± 3.2, 57.2 ± 7.1, 72 ± 14.3, 58.9 ± 11.9 in the presence of 0.5, 2.5, 10, and 100 μM Y27632 respectively. The absence of change in cell spreading even as diphospho-MLC signal decreased suggests that at least in this cell type, MLC diphosphorylation is not directly responsible for cell spread area. It should be noted that even though the cell spread area did not change with drug treatment, there appeared to be changes in cell shape and the roughness of cell edges. At the highest level of Y27632 treatment, 100 μM, there was a statistically significant increase in cell perimeter compared to untreated cells (p < 0.05, n = 3) (Fig. 3D). Images of cells in the absence of Y27632 (Fig. 3E), 10 μM Y27632 (Fig. 3F) and 100 μM Y27632 (Fig. 3G) are shown.

**Figure 4:**
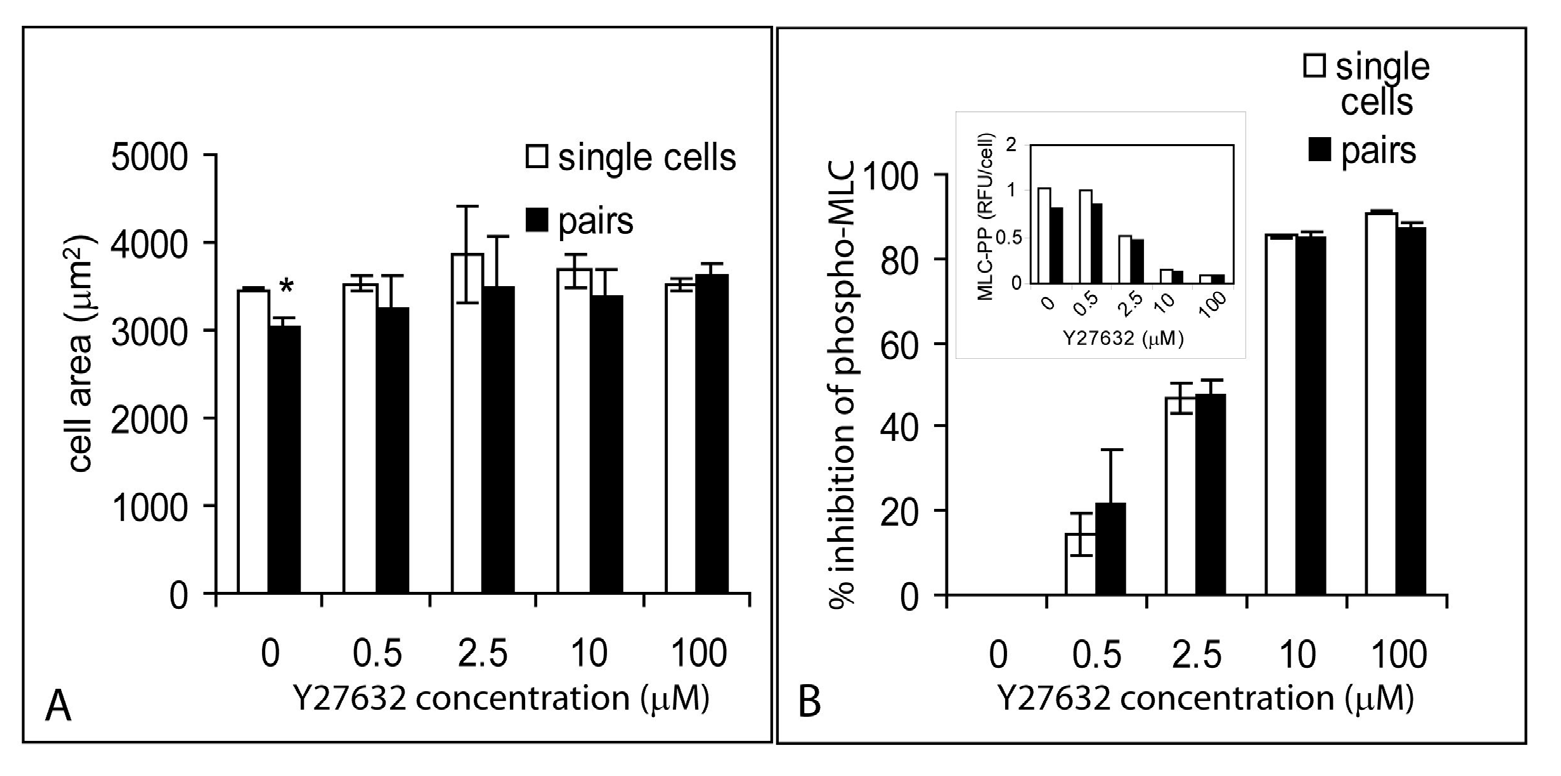
**Comparison of phospho-MLC response in individual cells with cells in contact with other cells**. Means and standard deviations (n = 3 replicate experiments) are shown. Number of cells measured is about 150 single cells and 30 cells in pairs. A) Comparison of cell spreading between individuals not in direct physical contact with other cells and pairs of cells contacting each other. Statistical significance (p < 0.05) is indicated by '*'. B) Comparison of the percent inhibition of MLC phosphorylation by Y27632 between individuals not in direct physical contact with other cells and pairs of cells contacting each other. Inset B: comparison of absolute MLC phosphorylation in response to Y27632 in single cells and pairs from a representative experiment (Y-axis scale: RFU × 10^6^).

In the analysis presented thus far, we selectively considered single cells that were not in direct contact with other cells. The automated microscopy and staining techniques employed in this study allow us to determine if MLC diphosphorylation levels in single cells are different from levels in cells that are in contact with each other. Cell-contact mediated through cadherins has been reported to change RhoA activity in several different cell types [16,17]. Since RhoA is an upstream activator of ROCK, we examined possible downstream effects of cell-cell contact on cell spreading and MLC diphosphorylation. In the absence of Y27632 the average spread area per cell of pairs of cells that contacted each other appeared to be significantly less than that of isolated single cells (Fig. 4A, p < 0.01, n = 3). However, this difference was not observed upon treatment with Y27632 (Fig. 4A, p > 0.1, n = 3). Furthermore, there was no apparent difference in MLC diphosphorylation per cell in pairs compared to single cells at any of the concentrations of Y27632 used (Fig. 4B). We conclude that under these conditions, cell-cell contact does not affect MLC diphosphorylation in vascular smooth muscle cells.

**Figure 5:**
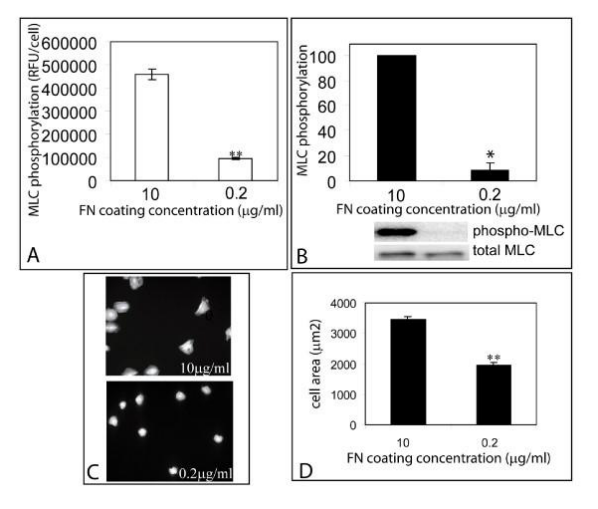
**Phospho-MLC response to ECM density**. Comparison of MLC phosphorylation (A, B) and cell spreading (C, D) between cells on substrates coated with 10 μg/ml FN and 0.2 μg/ml FN. Error bars indicate standard deviations of 3 replicate experiments. Statistical significance (p < 0.005) is indicated by '**', (p<0.05) by '*'.

To further validate the method, we employed additional ways to perturb myosin light chain phosphorylation. ECM density influences myosin light chain phosphorylation by changing the functional coupling between Rho and ROCK (Bhadriraju et al, 2007). Hence we examined the effect of lowering ECM density on MLC phosphorylation. The coating concentration of fibronectin was lowered from 10 μg/ml to 0.2 μg/ml. Myosin phosphorylation on 0.2 μg/ml FN surfaces was 20.7% ± 1.44% of controls (10 μg/ml fibronectin) as measured by microscopy (Fig. 5A) and 8.47% ± 5.51% of controls as measured by western blotting (Fig. 5B). Correspondingly, lowering ECM density also reduced the extent of cell spreading (Fig. 5C, D).

**Figure 6:**
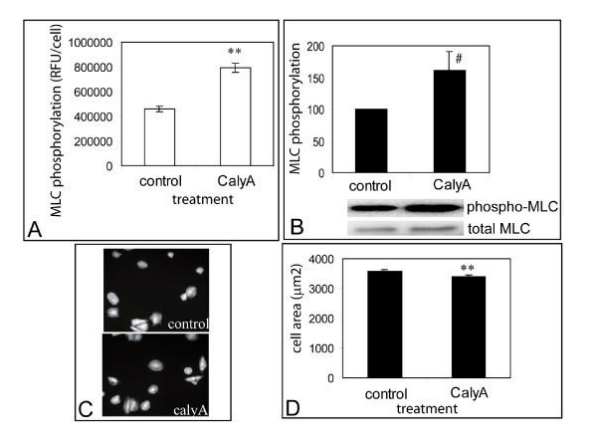
**Phospho-MLC and cell spreading in response to the addition calyculin A**. Comparison of phospho-MLC (A,B) and cell spreading (C, D) in response to the addition of 5 ng/ml of calyculin A. Error bars indicate standard deviations of 3 replicate experiments. Statistical significance (p < 0.005) is indicated by '**', # (p = 0.057).

We also examined the effect of perturbations designed to increase MLC phosphorylation relative to controls. Cells were treated with the protein phosphatase inhibitor calyculin A to activate MLC phosphorylation, and MLC phosphorylation was measured by microscopy and western blotting. Addition of 5 nM calyculin A to cells for 3 minutes increased MLC phosphorylation to 172.1% ± 7.42% of untreated controls as measured by microscopy (Fig. 6A) and 160.5% ± 30.1% of controls as measured by western blotting (Fig. 6B). There was a small but statistically significant decrease in cell area within 3 minutes after drug addition detected by quantitative microscopy (Fig. 6C, D). It was observed that cells completely rounded up at approximately 10 minutes after the addition of the drug (data not presented).

**Figure 7:**
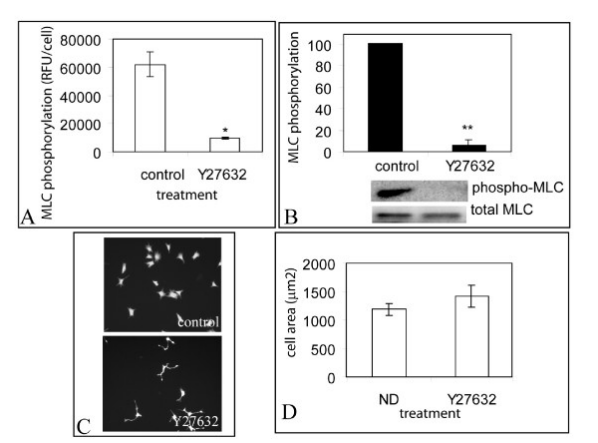
**Measurement of ML phosphorylation in NIH 3T3 cells**. A) Quantitative Microscopy measurement of phospho-MLC in the absence or in   the presence of 10 μM Y27632 in 3T3 cells, B) corresponding western blotting   data and C,D) cell spreading. Error bars indicate standard deviations of 3   replicate experiments. Statistical significance (p < 0.005) is indicated by   '**'. * (p<0.05).

We finally examined if the method could measure MLC phosphorylation in other cell types by using Y27632 to inhibit MLC phosphorylation in NIH 3T3 cells. 10 μM Y27632 was found to inhibit MC phosphorylation by 85% relative to non-drug treated controls, both by quantitative microscopy and western blotting (Fig. 7A,B) without a statistically significant change in cell area (Fig. 7C, D).

## Discussion

We report here a robust protocol for quantitative immunofluorescence microscopy, where cell fixation, staining and imaging were optimized for quantitatively analyzing both cell morphology and MLC phosphorylation levels within individual adhered cells. This approach allows evaluation of the relationship between the two parameters. The use of careful consideration of staining protocols can quantitatively preserve phosphorylated proteins, as well as intracellular spatial information, and allows us to distinguish extent of phosphorylation in single cells from cells that are in contact with other cells.

Using imaging cytometry, we determined that Y27632 inhibits MLC diphosphorylation with an IC_50 _of about 2.1 ± 0.6 μM in A10 vascular smooth muscle cells, which was not significantly different from the value determined by Western blotting under identical conditions. Experiments measuring contractility response in vascular and bronchial smooth muscle strips indicated an IC_50 _between 0.3 – 1 μM for Y27632 [18]. Another study measuring the inhibition of neutrophil migration as a function of Y27632 concentration reported an IC_50 _value between 3–10 μM [19]. The correspondence between these sets of data indicates that the microscopy-based assay presented here likely provides an accurate determination of the level of diphospho-MLC in cells.

We tested the validity of the technique used here in a number of ways. We directly compared different fixation protocols for efficacy of retaining intracellular structure and phospho-MLC signal. We examined two different cell types: A10 vascular smooth muscle cells, and NIH 3T3 cells, and compared average MLC phosphorylation measured by imaging cytometry to that determined by western blotting. We used several methods to perturb phospho-MLC levels including the use of Y27632 to inhibit Rho kinase (Fig. 2, 7), plating cells on a low ECM density (Fig. 5), and treating cells with an activator of contractility, calyculin A to increase MLC phosphorylation (Fig. 6). In all cases, we found that measurement of MLC phosphorylation by microscopy corresponded to the same measurements by western blotting.

Two enzymes are known to be principally responsible for activation of MLC by phosphorylation, myosin light chain kinase (MLCK) and Rho-associated kinase (ROCK). Since cytoskeletal tension is dependent partly on changes in MLC phosphorylation, pathways, and smooth muscles play an important role in regulating vascular tone, drugs regulating the latter have been the focus of drug discovery efforts for vascular therapies [12]. In this study we have used the ROCK inhibitor, Y2763, and have quantified resulting inhibition of diphosphorylation of MLC. Other reports have suggested that blocking MLCK reduces MLC phosphorylation and cell spreading [20,21], but blocking ROCK, which also inhibits MLC phosphorylation, does not reduce cell spreading [21]. We found that blocking ROCK does not appear to influence cell spread area, even though it quantitatively inhibits diphosphorylation of MLC. Myosin light chain phosphorylation may be one of the variables that regulates cell spreading. Other elements of the cytoskeleton such as actin, tubulin, linker and scaffolding proteins in focal adhesions may also be expected to play a role in the spreading process.

By simultaneously measuring cell morphology and corresponding MLC diphosphorylation levels in individual cells, we find that there is a linear relationship between cell spread area and the amount of MLC diphosphorylation. While the addition of the ROCK inhibitor Y27632 decreases the total amount of MLC diphosphorylation it does significantly affect the linear relationship between the two variables, and does not affect the range of cell spread areas observed across the population (Fig. 3C). We do measure subtle changes in cell shape, with an increase in roughness of the perimeter of cells, in the presence of 100 μM Y27632 (fig. 3D). As demonstration of the topographical information that can be simultaneously extracted uniquely by quantitative microscopy, we compared myosin light chain phosphorylation between single cells not in contact with each other compared to pairs of cells in contact with each other measured by microscopy (Fig. 4). The absence of difference on MLC phosphorylation between singles and pairs of cells suggests that cell-cell contact may not regulate MLC phosphorylation in this cell type.

The advantages of imaging cytometry compared to a technique such as western blotting are many: imaging provides additional information such as variation in response over the population and allows correlation of different parameters within individual cells. However the results of imaging cytometry are only useful if they result from validated protocols that provide accurate information about intracellular components. We hope that studies such as this will help to guide the process of critical evaluation of protocols as well as provide useful protocols for more accurately quantifying imaging results.

## Conclusion

The results presented here indicate that it is possible to accurately measure phosphorylation of myosin light chain in large numbers of individual cells using automated immunofluorescence microscopy, provided that adequate care is taken to validate fixation, staining and quantification protocols. The morphological information afforded by microscopy can be coupled with quantitative intensity information to study phosphorylation events in cell signaling in relation to other cellular parameters. Using this technique it should be possible to screen pharmaceutical compounds for their effects on myosin activity-based cell behaviors such as cell spreading, growth, and migration.

Importantly, this study shows that measurements on individual cells can reveal trends that population-averaged measurements can mask. One important observation is that the different levels of phospho-myosin measured in individual cells constitute a reproducible distribution of responses across the cell population, suggesting the stochastic nature of the phosphorylation reaction.

## Methods

### Cell culture

Rat aortic smooth muscle cell line A10 (vSMC; ATCC, Manassas, VA), or NIH 3T3 cells, were cultured in Dulbecco's Modified Eagles Medium (DMEM; Mediatech, Herndon, VA) DMEM supplemented with 10% (v/v) fetal bovine serum and non essential amino acids, glutamine (4 mM) (all from Gibco Invitrogen, Carlsbad, CA), and maintained in a humidified 5% (v/v) CO_2 _balanced-air atmosphere at 37°C. Fibronectin (BD Biosciences, Franklin Lakes, NJ) was coated on to substrates by incubating 24-well bacteriological plates (Nalgene Nunc, Rochester, NY) with 10 μg/ml of human plasma fibronectin (BD Biosciences) in phosphate buffered saline (PBS) for 30 minutes. The plates were then treated with 1% (w/v) bovine serum albumin (BSA) in PBS for 30 minutes to reduce nonspecific adsorption of proteins from the cell culture medium. Cells were plated at a density of 2000/cm^2^. For the ROCK inhibition experiments, serial dilutions of Y27632 (Tocris, Ellisville, MO) were made in water, and all cells received equal volume of the diluent (1 μl in 500 μl media) at the final drug concentration. The drug was added 2 h after plating cells and samples were fixed at 4 h. For culture on a matrix with lower adhesiveness, plates were coated with 0.2 μg/ml of fibronectin in PBS. For the experiments employing calyculin A, the drug dissolved in DMSO was added at a concentration of 5 ng/ml for 3 minutes and cells were processed for myosin phosphorylation measurement.

### Cell fixation, permeabilization, and staining procedures

To minimize enzymatic activity, cells were exposed to fixative solution immediately after removal from the incubator (typically within 1 minute). For fixation and simultaneous permeabilization with methanol, ethanol, and acetone, media was removed and ice-cold solvent was added for 20 minutes in the refrigerator. Solvent was removed and the sample was rinsed three times. For fixation or simultaneous fixation/permeabilization with paraformaldehyde (PFA; Electron Microscopy Sciences, Hatfield, PA), media was removed and replaced with 4% (v/v) PFA in PBS, or 4% PFA+ 0.5% Triton X-100 (v/v) in PBS, at 37°C, and samples were maintained at room temperature for 20 minutes. The cells were then rinsed thrice with PBS over a 5-minute interval followed by two rinses with TBS (Tris-buffered saline) over another 5 minutes. When they were not simultaneously permeabilized with Triton X-100 during the fixation process, PFA fixed cells were rinsed as above and permeabilized by adding 0.05% Triton X-100 in PBS (PBSTX) for 10 minutes. Alternatively they were rinsed and permeabilized by adding chilled methanol, ethanol, or acetone respectively, for 10 minutes followed by rinsing 3 times with PBSTX. The fixed and permeabilized cells were processed immediately for staining.

Cells were first stained with Texas-Red maleimide (Tx-Red) to provide good discrimination of the cell area [15] by incubating with a 1:10^6 ^(v/v) solution of Tx-Red (stock 10 mg/mL in DMF) in PBS on a shaker with gentle side-to-side shaking for 30 minutes. Sample were rinsed 5 times with PBSTX over a 10 minute period and immediately subjected to immunofluorescence staining. Cells were incubated with a blocking buffer (10%(v/v) goat serum + 1%(w/v) BSA in PBS) for 20 minutes, switched to 1:100 dilution of anti-diphospho MLC in blocking buffer (rabbit polyclonal anti-diphospho MLC; Cell Signaling Technologies, Boston, MA) for 45 minutes, rinsed five times with PBSTX, reincubated with blocking buffer for 10 minutes, switched to 20 μg/ml of Alexa 488 goat anti-rabbit antibody (Invitrogen Corporation, Carlsbad, CA) in PBS and 1 pg/ml of DAPI in 10% glycerol/PBS (v/v) for 45 minutes. In the experiments where stress fibers were stained, TRITC-phalloidin (Invitrogen) at 33 nM final concentration was added to the solution containing the secondary antibody. Cells were finally rinsed five times with PBSTX and switched to mounting medium (90% (v/v) glycerol in Tris pH 8.0 + 0.25% (w/v) DABCO (Sigma, St. Lois, MO)).

### Microscopy and Image Analysis

A Zeiss Axiovert 100 inverted fluorescent microscope fitted with a computer-controlled automated stage and color filter wheels, dichroic beamsplitter (set# 84000; Chroma Technology Inc., Brattleboro, VT), phase microscopy optics, a 100 W mercury arc lamp, Coolsnap FX CCD camera (Roper Scientific/Photometrics, Tucson, AZ), and ISee image acquisition software (Isee Imaging Systems, Rayleigh, NC) was used for image collection. The various color filters (all from Chroma Technology Inc., with the peak wavelength/width, and Chroma part numbers as indicated) that were used for detecting the different fluorophores are as follows: DAPI (Excitation 360/40, part number #38086; Emission 460/50, part number #42919), Alexa 488 (Excitation 470/40, part #51359; Emission 525/50, part # 42345), Tx-Red (Excitation 555/28, part #32295; Emission 630/60, part #41834). For high resolution color images, cells were seeded on fibronectin-coated glass coverslips, stained as described above and imaged under a 40 × objective. Overlays of images taken with different optical filters shown in Figure 1 are presented without any readjustment of the brightness or contrast in the individual color channels.

For all other data, a single protocol was used. For each sample, 50 fields were selected by automated stage movement and images were collected for Tx-Red fluorescence (for determining cell area), Alexa 488 (to measure diphospho-MLC), and DAPI (to identify the nucleus) using a 10 × objective lens. Images were analyzed using an analysis routine written for NIH ImageJ [22,23]to determine spread area for each Tx-Red stained object, the presence of and the number of nuclei (to confirm that each detected object was one or more cells), and to integrate the immunofluorescence intensity within each cell object. For background subtraction, a parallel set of samples was similarly fixed and stained, except there was no primary antibody incubation step. We plotted cell area vs. the integrated background signal obtained from secondary antibody-only controls and fitted the plot to a straight line to obtain the background signal as a function of cell area. Intensity appropriate to cell area was then subtracted from the measured phospho-MLC signal for each cell. Tx-Red fluorescence did not contribute to fluorescence in the Alexa 488 channel as determined by the low correlation coefficient seen for plots of Tx-Red fluorescence intensity vs. Alexa 488 fluorescence intensity (data not shown).

### Western Blotting

To measure relative MLC-phosphorylation by immunoblotting, dishes with cells were placed on ice and gently rinsed with ice-cold PBS, directly lysed into chilled SDS-PAGE sample buffer and immediately boiled for 5 minutes. Samples were stored at -80°C when not used immediately. Lysates were separated on 4–20% (w/v) gradient polyacrylamide gels (ReadyGels™, Biorad, Hercules, CA) and separated proteins were transferred onto PVDF membrane (Biorad). The PVDF membranes were then blocked with 6% (w/v) non-fat dry milk. Blots were probed with the antibody against diphospho-MLC, and the signal was normalized for that of total myosin using an antibody against myosin light chain (Sigma-Aldrich, St Louis, MO). Bands were detected using horse radish peroxidase-labeled secondary antibodies and chemiluminescence detection (Pierce, Rockford, IL), on a Genegnome imager (Syngene, Frederick, USA).

### Data Analysis

IC_50 _was determined by fitting the inhibition of phospho-MLC relative to the no-drug condition, to the equation y = a*(1-exp(-b*x)) where y is the percent inhibition of phospho-MLC and x is the concentration of Y27632, using BioDataFit 1.02 [24] and the fit was verified using Microsoft Excel. Statistical significance was ascertained using Student's t-test. For Western blot data and for image data, percent inhibition was determined from the ratio of band density or immunofluorescence signal in the presence of Y27632 to signal in the absence of Y27632, and subtracting from 100.

## Competing interests

The author(s) declares that there are no competing interests.

## Authors' contributions

KB and MN performed the experiments. JTE provided protocols for automated microscopic analysis. KB, JTE and ALP performed data reduction and analysis, manuscript preparation. All authors read and approved the final manuscript.
